# Check and mate to exosomal extracellular miRNA: new lesson from a new approach

**DOI:** 10.3389/fmolb.2015.00011

**Published:** 2015-04-13

**Authors:** Andrey Turchinovich, Alexander G. Tonevitsky, William C. Cho, Barbara Burwinkel

**Affiliations:** ^1^Molecular Epidemiology, C080, German Cancer Research Center (DKFZ)Heidelberg, Germany; ^2^Molecular Biology of Breast Cancer, Department of Gynecology and Obstetrics, University Clinic HeidelbergHeidelberg, Germany; ^3^Hertsen Federal Medical Research Centre of the Ministry of Health of the Russian FederationMoscow, Russia; ^4^Department of Clinical Oncology, Queen Elizabeth HospitalHong Kong, China

**Keywords:** miRNA, exosomes, microvesicles, cell-cell communication, argonaute proteins, nanoparticle tracking analysis

## Abstract

MicroRNAs (miRNAs) are 19–24 nt single-stranded RNAs which regulate gene expression by sequence-specific targeting of corresponding mRNAs. Extracellular miRNAs have been consistently detected in all human body fluids, and were shown to be prominent non-invasive biomarkers for various diseases including cancer. Albeit biological function of cell-free miRNA remains questionable, some studies demonstrated that exosomes encapsulated extracellular miRNAs could mediate inter-cellular signaling. While others suggested that these miRNAs are mostly by-products of cellular activity and do not carry any significant biological function. This article aims to discuss the current theories of origin of extracellular miRNA, and to highlight recent application of a novel technique of micro-vesicles counting, that may challenge the existence of exosomal miRNA.

The number of reports addressing extracellular circulating miRNA has exploded in recent years, revealing enormous interest in this emerging field. Extracellular miRNAs have been reported to participate in a wide range of processes implying both short range and distant cell-cell communication between various healthy and cancer cell types (Valadi et al., 2007; Skog et al., 2008; Kosaka et al., 2010; Pegtel et al., 2010; Kogure et al., 2011; Mittelbrunn et al., 2011; Chen et al., 2012; Hergenreider et al., 2012; Montecalvo et al., 2012; Xin et al., 2012). Multiple observations that extracellular circulating miRNAs could be exported by cancer cells suggested their use as promising non-invasive biomarkers of tumorigenesis (Taylor and Gercel-Taylor, 2008; Rabinowits et al., 2009; Ohshima et al., 2010; Cho, 2011; Cortez et al., 2011). However, the biological significance of the miRNA encapsulated in extracellular vesicles (EV) is still unclear (Turchinovich et al., 2012).

Lawrie and colleagues pioneered the discovery of miRNAs in biological fluids while investigating several tumor-associated miRNA species in serum samples from diffuse large B-cell lymphoma patients (Lawrie et al., 2008). They also suggested for the first time that miRNA have potential as non-invasive biomarkers for cancer diagnosis and prediction of the outcome. However, it remained unclear whether miRNAs are derived from tumor cells or resulted from indirect response to cancer. In the same year, Tewari's group showed that both host and tumor derived cell-free miRNA were present in plasma and serum in a remarkably stable form (Mitchell et al., 2008). While the exact mechanism which rendered the stability of circulating miRNA was not addressed, the observation that many miRNAs were co-purified with exosomes and microvesicles exported by cultured cells (Valadi et al., 2007) provoked the hypothesis that cell-free miRNA in plasma is protected by the EV membranes. Furthermore, Hunter and colleagues detected miRNAs in the fractions of purified human peripheral blood microvesicles that reinforced the above hypothesis (Hunter et al., 2008). Along with some reports that the exchange of miRNA (and also mRNA) between cells can be accomplished through exosome-mediated transfer, these findings led to a revolutionary paradigm—the existence of inter-cellular and inter-organ communication by means of EV-encapsulated miRNAs. Nevertheless, neither report revealed the existence of EV-free extracellular miRNA at that time.

Unexpectedly, the assumption that only EV-encapsulated miRNAs are present in biological fluids was challenged by two simultaneous independent studies published in 2011 and demonstrating that 95–99% of extracellular miRNA are not only EV-free but also associated with proteins of the argonaute (AGO) family, in plasma/serum and cell culture media (Arroyo et al., 2011; Turchinovich et al., 2011). Indeed, during intracellular miRNA synthesis all mature miRNAs become associated with one of the AGO proteins (Bartel, 2004) and, therefore, it was expected that extracellular miRNA can be coupled with the same proteins. Furthermore, the association of Argonaute-bound miRNAs with both exosomes and microvesicles could in theory be explained by the known ability of RNA-binding proteins to efficiently bind the membranes (Agapov et al., 1997; Pohl et al., 1998). Consistently, the remarkable stability of AGO2 protein explained the resistance of associated miRNAs in cells-free nuclease and protease rich environment (Turchinovich et al., 2011). None of the studies has addressed the copy number of the remaining 1–5% of extracellular miRNA fractions which was indeed co-purified with exosomes and microvesicles. Nevertheless, it became apparent that the vast majority of the extracellular miRNAs cannot mediate cell-cell communication function directly, as they were solely AGO-proteins bound by-products of cell death (Arroyo et al., 2011; Turchinovich et al., 2011).

A recent study by the Tewari group has significantly challenged the “exosomal miRNA hypothesis” as it revealed that most individual exosomes in standard preparations do not carry biologically significant numbers of miRNAs (Chevillet et al., 2014). The authors used a novel nanoparticle tracking analysis (NTA) method to quantify the abundance of extracellular exosomes, and compared it with the molarity of accompanied miRNAs. They quantified both exosomes and miRNA molecules number within plasma, seminal fluid as well as conditioned media from dendritic cells, mast cells, and ovarian cancer cells. Strikingly, in all analyzed samples the ratio of miRNA molecules to exosomes was significantly less than one. More strikingly, on average, 100 exosomes corresponded to only one copy of a given miRNA. In previous reports, the quantification of extracellular exosomes has been performed by manual counting after visualization using transmission electron microscopy (TEM) (Thery et al., 2006; Valadi et al., 2007; Montecalvo et al., 2012). However, due to complicate sample preparation process, TEM is not a “quantitative” method. On the contrary, the NTA approach allows precise quantification of exosomes under 300 nm after visualization of light scattering induced by the particles using a light microscope (Dragovic et al., 2011; Chevillet et al., 2014). The fact that most individual exosomes do not carry a biologically significant number of miRNAs strongly suggest that extracellular miRNA cannot mediate cell-cell communication by direct targeting mRNAs in the recipient cells. However, the impact of the protein protected miRNAs on extracellular receptors cannot be excluded.

Since Chevillet and colleagues only investigated exosomal fractions of the extracellular fluids, their study did not exclude a possibility that EVs larger than exosomes (e.g., microvesicles) may contain more copies of miRNA. However, the removal of microvesicles by either ultra-filtration or high-speed centrifugation does not alter the overall levels of extracellular miRNA in both cell-free plasma and conditioned media, indicating that microvesicles may contain either similar or smaller amounts of miRNA than exosomes (Turchinovich et al., 2011). More importantly, the assumption that some miRNAs are incorporated into exosomes and microvesicles were so far solely based on observations that EV and certain miRNAs co-purify by sedimentation or/and ultrafiltration. It remains to be tested whether EV-free extracellular miRNA in forms of large protein aggregates (e.g., fully assembled RISC) co-purifies with exosomes.

Beyond cell-cell communication, the diagnostic and disease prognostic potential of extracellular miRNA is still a very valid area. Indeed, during toxicity in certain organs and tissues (Laterza et al., 2009; Corsten et al., 2010; Zhang et al., 2010), AGO proteins associated miRNAs are released into the extracellular space, and travel to distant parts of the body due to the incredible stability of AGO-miRNA complex (Turchinovich et al., 2011, 2013; Turchinovich and Burwinkel, 2012). Thus, in patients with chronic hepatitis B, the increase of liver specific miR-122 in the plasma strongly correlated with the severity of the disease. More importantly, compared with an increase in aminotransferase activity (a common marker for liver damage) in the blood, the increase in miR-122 concentration appeared earlier (Zhang et al., 2010). Likewise, dying tumor cells or immune cells during inflammation can alter the “healthy” pattern of extracellular miRNAs. Indeed, many cancer-tissue-specific miRNAs and “inflammatory” miRNAs have been found in the blood circulation at different stages of the disease (Mitchell et al., 2008; Skog et al., 2008; Rabinowits et al., 2009; Cho et al., 2011; Olivieri et al., 2013). However, the diagnostic promised of cell-free miRNA has to be regarded with carefulness. Thus, Leidner and colleagues have failed to reproduce breast cancer predictive values of many circulating miRNAs in a genome-wide plasma miRNA profiling experiment (Leidner et al., 2013). In addition, there is a significant inconsistency among multiple previous studies demonstrating cancer-associated miRNA patterns in biological fluids, highlighting the need for better standardization of pre-analytical factors, samples processing, miRNA profiling methods and data analysis.

In light of the recent evidences the concept that human cells export extracellular miRNA packed into EV has to be thoroughly validated (Chevillet et al., 2014). While the presence of other RNA species including tRNA, rRNA, snRNA, snoRNA, Y RNA in the micro-vesicles and exosomes cannot be yet excluded, the current paradigm of extracellular miRNA has to be revised and updated.

## Conflict of interest statement

The authors declare that the research was conducted in the absence of any commercial or financial relationships that could be construed as a potential conflict of interest.
